# Supplementary material for: Analysis of Sequence Divergence in Mammalian ABCGs Predicts a Structural Network of Residues That Underlies Functional Divergence
Source: Int J Mol Sci. 2021 Mar 16;22(6):3012. doi: 10.3390/ijms22063012 (PMC8001107; doi:10.3390/ijms22063012)
Supplement: Supplementary file 1 [file ijms-22-03012-s001.zip › Supplements/Conservation patterns with refs.pdf]

(ABCG1), (ABCG4), (ABCG2), (ABCG5), (ABCG8)

Alignment position	ABCG2	ABCG5	ABCG8	References	Summary of recorded effects
362	K47	S48	D57		
749	H267	C274	L294		
909	P392	K383	L414		
1192	K653	D646	Q667	Khunweeraphong et al 2017	ABCG2: protein immature

(ABCG1, ABCG4), (ABCG2), (ABCG5, ABCG8)

Alignment position	ABCG2	ABCG5	ABCG8	References	Summary of recorded effects
539	N139	T147	T166		
676	T194	V201	V221		
683	I201	L208	L228		
729	Y247	S254	S274		
755	A273	M280	M300		
770	N288	S295	S315		
780	I298	T305	T325		
928	A411	F402	F433		
957	C438	P431	P460		
976	K452	R446	R475	Khunweeraphong et al 2017	ABCG2: diminished mature expression, reduced mitoxantrone transport. Elsewhere, multiple nonsense mutations of ABCG5.
986	S461	D455	D484		
988	Y463	L457	L486		
1103	I573	F567	F595	Haider et al 2015	ABCG2: protein immature

(ABCG1, ABCG4), (ABCG2), (ABCG5), (ABCG8)

Alignment position	ABCG2	ABCG5	ABCG8	References	Summary of recorded effects
514	G122	S130	A149		
680	M198	A205	V225		
684	T202	Q209	W229		
730	S248	E255	D275	Tamura et al 2006, Tamura et al 2007, Deppe et al 2014	ABCG2: reduced porphyrin transport, reduced resistance (MX, Dox, DNR, etoposide)
739	T257	A264	L284		
768	A286	E293	R313		
784	S302	T309	R329		
901	S384	V375	Q406	Polgar et al 2009	ABCG2: No expression with R383G background
912	S395	V386	L417		
914	A397	T388	I419		
918	V401	Q392	E423	Miettinen et al 2006	ABCG8: allelic variant

919	T402	N393	A424	Cox et al 2018, Ni et al 2010, Polgar et al 2010	ABCG2: reduced transport
941	G422	A415	S444		
953	T434	V427	G456	Heyes et al 2018, Toyoda et al 2019	ABCG2: transport abolished
954	T435	G428	A457		
959	S440	T433	N462	Cox et al 2018	ABCG2: reduced transport (MX, Pheo A)
960	S441	G434	V463	Nakagawa et al 2008, Tamura et al 2006, 2007, Sjöstedt et al 2017	ABCG2: reduced expression, no porphyrin transport, reduced transport (Lucifer yellow, Estrone-1-Sulfate
961	V442	M435	I464		
984	Y459	S453	L482		
985	I460	Q454	E483		
993	S468	Q462	P491		
1005	S476	H470	G499	Heyes et al 2018, Toyoda et al 2019	ABCG2: transport abolished
1011	R482	S475	H504	Various	ABCG2: gain of function; transport of non-native substrates
1050	S521	E514	R543	Lu et al 2001	ABCG8: allelic variant
1063	V533	P527	T555		
1072	T542	L536	N564		
1073	I543	L537	A565		
1075	F545	I539	Y567	Cox et al 2018	ABCG2: reduced transport (MX, Pheo A)
1076	V546	A540	N568	Xavier et al 2020	ABCG5: reduced ATPase with CHS
1077	F547	G541	S569		
1094	W564	P558	V586	Haider et al 2015	
1104	P574	Q568	L596	Berge et al 2000, Li et al 2001, Lu et al 2001	ABCG8: allelic variant
1163	G625	R619	P639		

Totally conserved

Alignment position	ABCG2	ABCG5	ABCG8	References	Summary of recorded effects
224	M1	M1	M1		
359	Y44	Y45	Y54	Abellan et al 2010, Krawczyk et al 2011, Hubacek et al 2001, many others	ABCG8
361	V46	V47	V56		
462	I78	I84	I103		
469	G80	G86	G105		
472	G83	G89	G108		
474	G85	G91	G110		

495	G104	G111	G130		
500	N109	N116	N135		
501	G110	G117	G136		
518	Q126	Q134	Q153		
520	D128	D136	D155		
534	L134	L142	L161		
535	T135	T143	T162		
536	V136	V144	V163		
538	E138	E146	E165	Khunweeraphong et al 2017, Keller et al 2011	ABCG2: protein immature, ABCG5: sitosterolemia
544	A144	A152	A171		
549	L146	L154	L173		
650	L168	L175	L195	Heimer et al 2002	ABG8: sitosterolemia
652	L170	L177	L197		
669	S187	S194	S214	Nakanishi et al 2003	ABCG2: reduced transport (DNR)
671	G189	G196	G216		
673	R191	R198	R218		
675	R193	R200	R220		
678	I196	I203	I223		
682	L200	L207	L227		
686	P204	P211	P231	Lee et al 2001, Lu et al 2001	
692	D210	D217	D237		
693	E211	E218	E238		
694	P212	P219	P239		
695	T213	R220	T240		Walker B motif
697	G215	G222	G242		
698	L216	L223	L243		
699	D217	D224	D244		
718	R236	R243	R263	Berge et al 2000, Lu et al 2001, Lee et al 2001	ABCG5: Nonsense mutations
725	H243	H250	H270		
726	Q244	Q251	Q271		ABCG8: Nonsense
727	P245	P252	P272		
732	F250	F257	F277		
735	F253	F260	F280		
736	D254	D261	D281		
744	G262	G269	G289	Keller et al 2011, Niu et al 2010	ABCG5: sitosterolemia
750	G268	G275	G295	Heyes et al 2018	ABCG2: allelic variant
763	G281	G288	G308		
766	C284	C291	C311		
771	N289	N296	N316		
772	P290	P297	P317		
774	D292	D299	D319		
775	F293	F300	F320		

900	R383	R374	R405	Polgar et al 2009, Khunweeraphong et al 2017, Lu et al 2001	ABCG2: poor expression, salt bridge with E458
907	G390	G381	G412		ABCG8: Nonsense
949	L430	L423	L452		
983	E458	E452	E481	Khunweeraphong et al 2017	ABCG2: poor expression, salt bridge with R383
989	Y464	Y458	Y487		
1009	P480	P473	P502	Cox et al 2018, Ni et al 2011	ABCG2: WT-like
1023	Y494	Y487	Y516	Cox et al 2018	ABCG2: reduced transport (MX)
1035	F506	F499	F528		
1054	L525	L518	L547		
1083	G553	G547	G575	Pandit et al 2006, Buch et al 2007	
1118	G588	G582	G610		
1171	L633	L627	L647	Cox et al 2018	ABCG2: protein immature
