# Supplementary material for: Analysis of Sequence Divergence in Mammalian ABCGs Predicts a Structural Network of Residues That Underlies Functional Divergence
Source: Int J Mol Sci. 2021 Mar 16;22(6):3012. doi: 10.3390/ijms22063012 (PMC8001107; doi:10.3390/ijms22063012)
Supplement: Supplementary file 1 [file ijms-22-03012-s001.zip › Supplements/Supplementary files revision.docx]

**Supplementary Table 1: Protein sequences used to identify functionally divergent positions.**


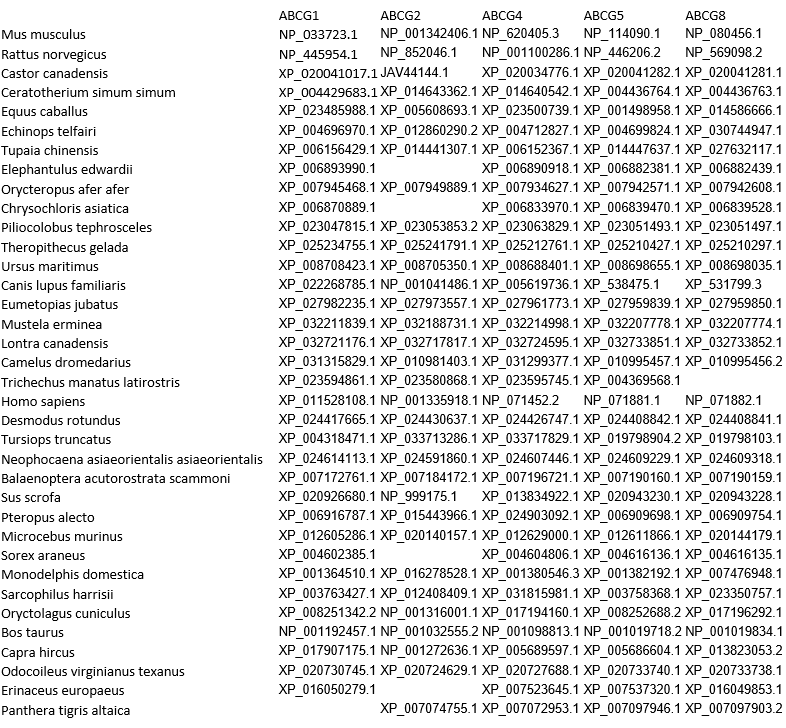

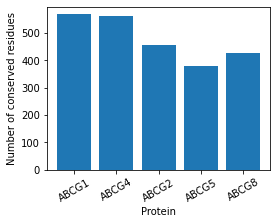


**Supplementary Figure 1: Overall alignment properties. a) Gap positions in the alignment.** 674 positions were excluded from further analysis as gaps as described in Methods. 595 positions were included, shown as dark lines in the figure. The NBD of ABCG2 is highlighted in red, and its TMD in blue. Arrows indicate (from left to right) the Q-loop, the signature motif, and the Walker A and B motifs. b**) The number of conserved residues for each protein.** This includes the 61 Totally Conserved positions and all functionally divergent residues.

**a)**

**b)**


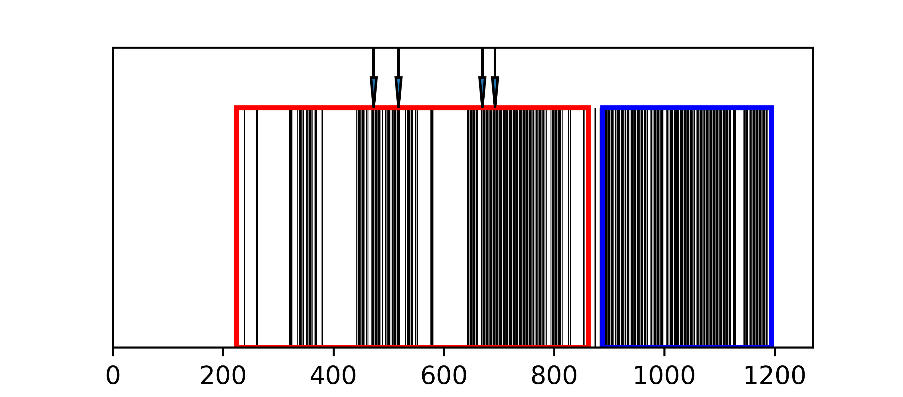


Position in alignment


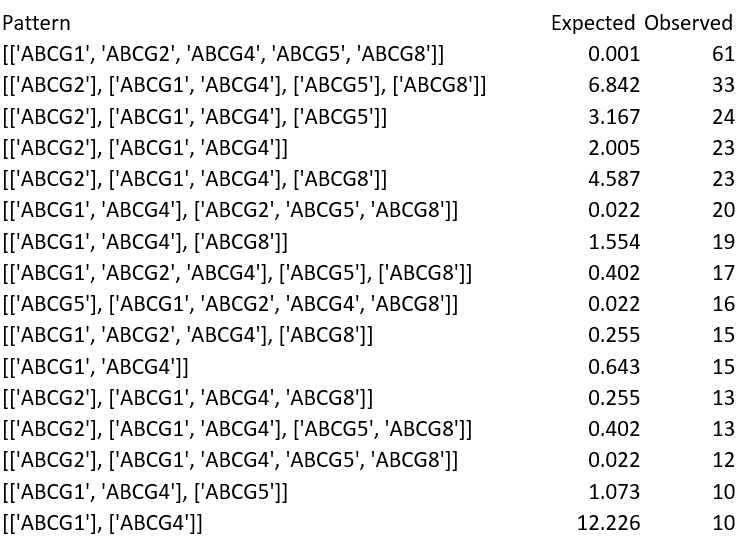
**Supplementary Table 2: Number of columns with a given conservation pattern across the whole alignment.** Only conservation patterns with 10 columns or more are included. Expected values (rounded to 3 decimal places) based on probabilities assuming conservation in each protein is independent of conservation in others.


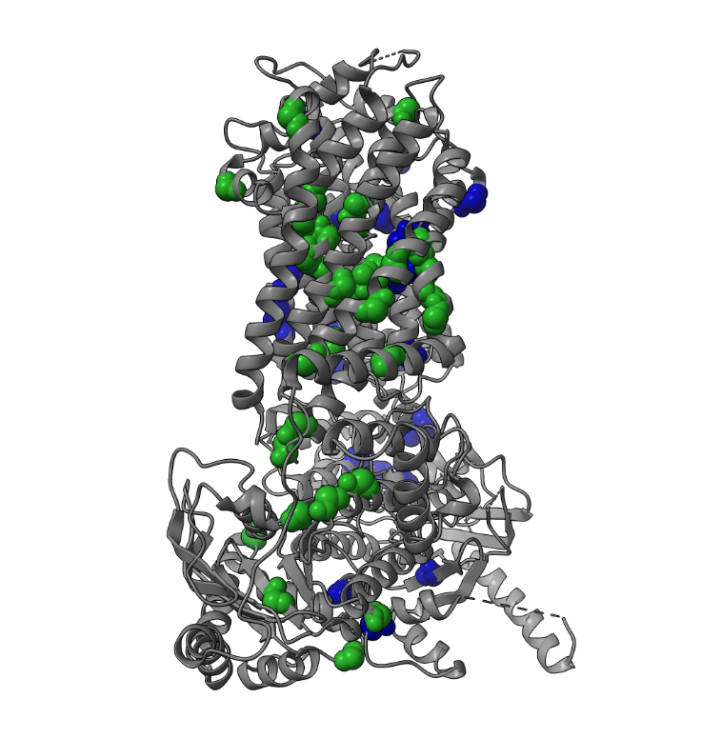

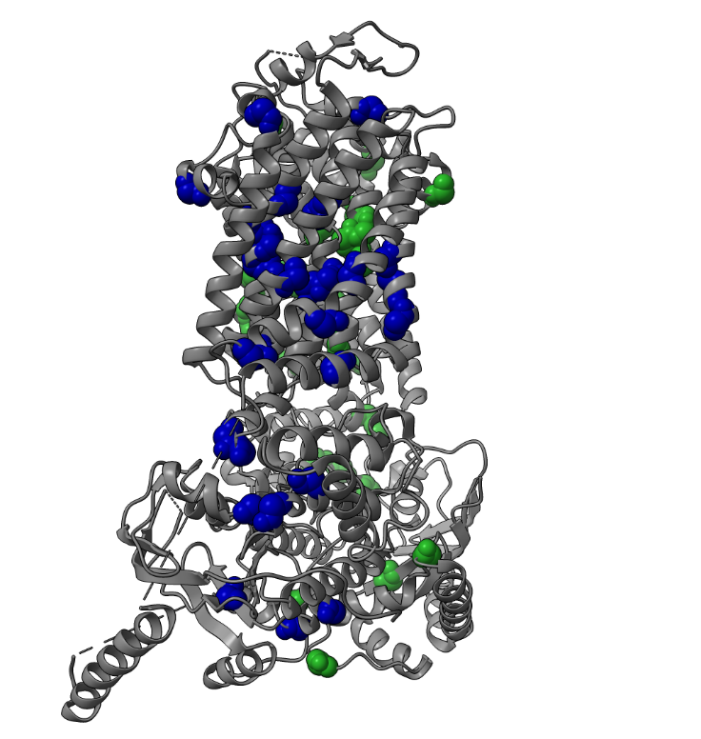

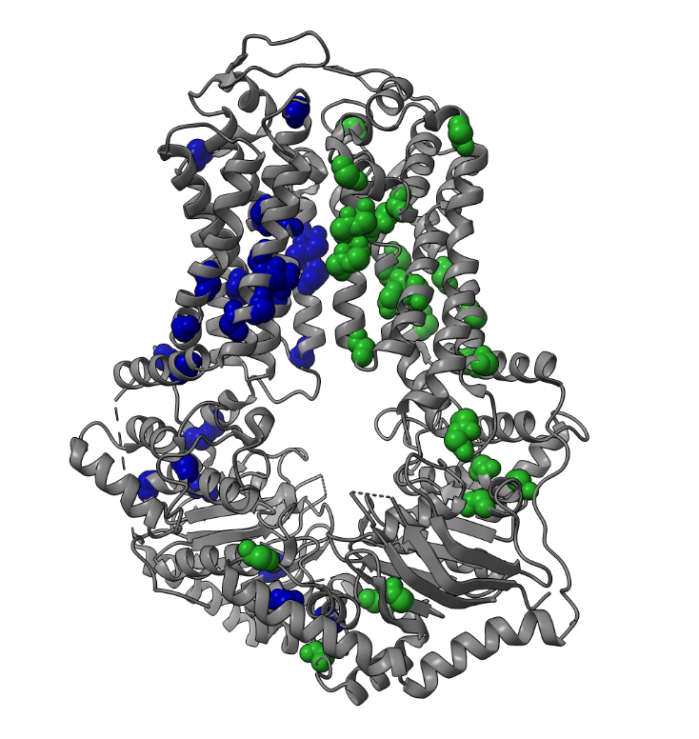


**Supplementary Figure 2: The conservation pattern (ABCG1, ABCG4), (ABCG2), (ABCG5), (ABCG8) on ABCG5/G8**. ABCG8 with blue spheres, ABCG5 with green spheres.

**Supplementary Table 3: Partial contingency table for conservation patterns in the polar relay.** Only conservation patterns represented in the polar relay are shown.


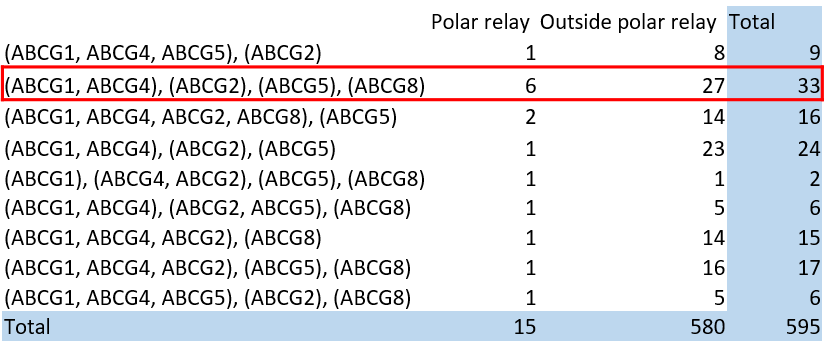

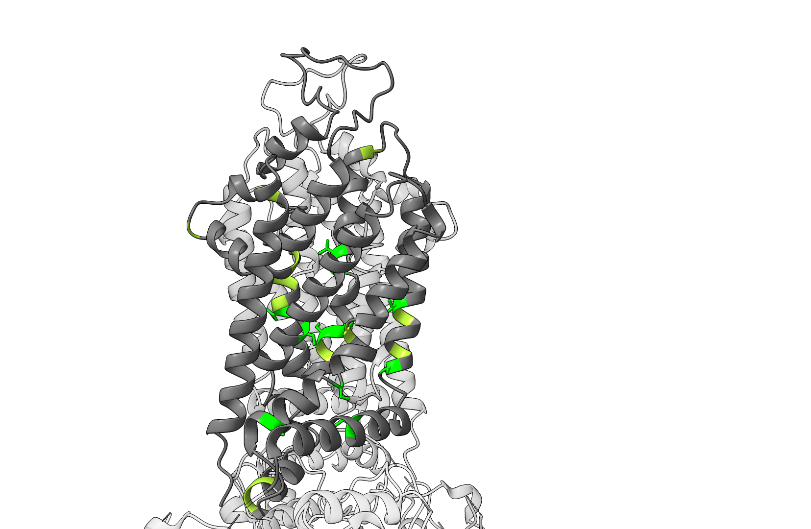

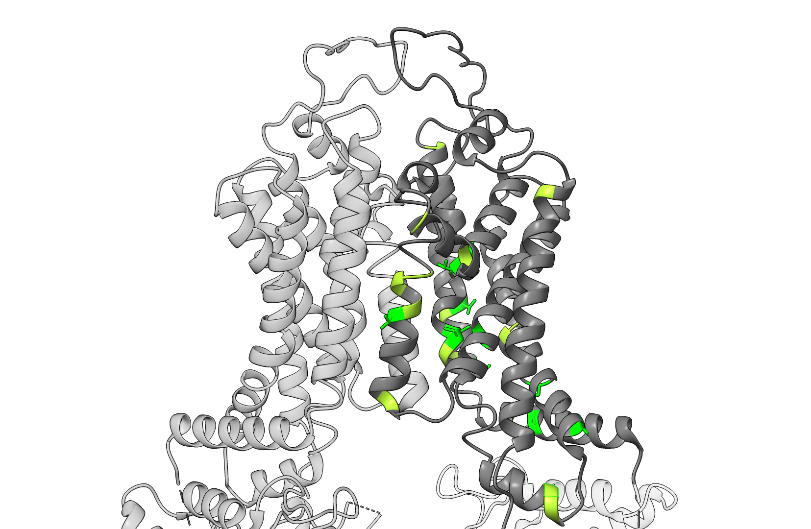

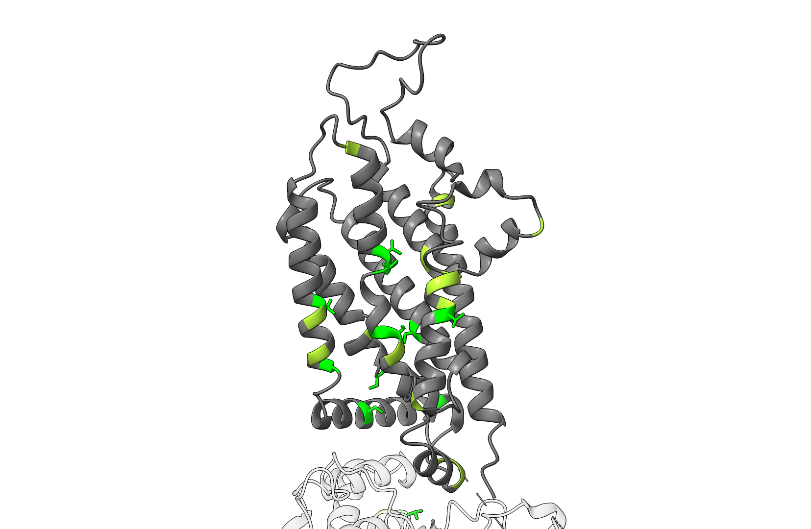


**a)**

**b)**

**c)**

**Supplementary figure 3: Serines and Threonines in the corkscrew** shown in lime green, both cartoon and stick representation. The rest of the residues in the TMD portion of the corkscrew are shown in yellow green. Views from the opposite face to the TMD-TMD interface in **a)**, the side in **b)** and the TMD-TMD interface in **c)**.


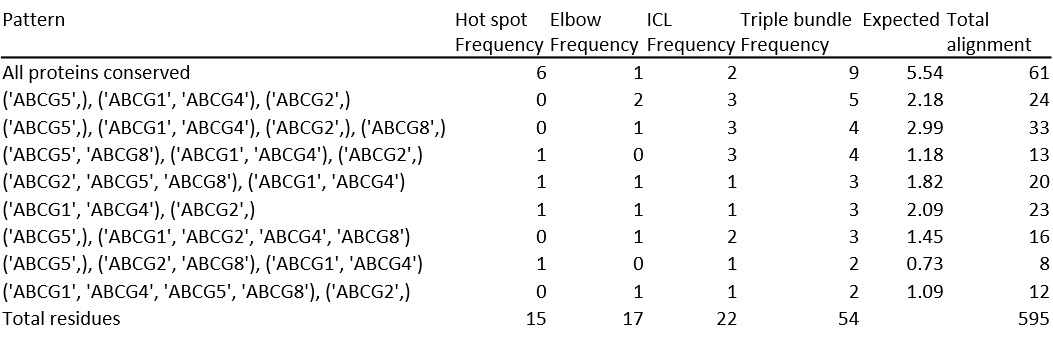
**Supplementary table 4: Frequency of conservation patterns in the triple helical bundle.** 18 conservation patterns with a single representative have been omitted for clarity.


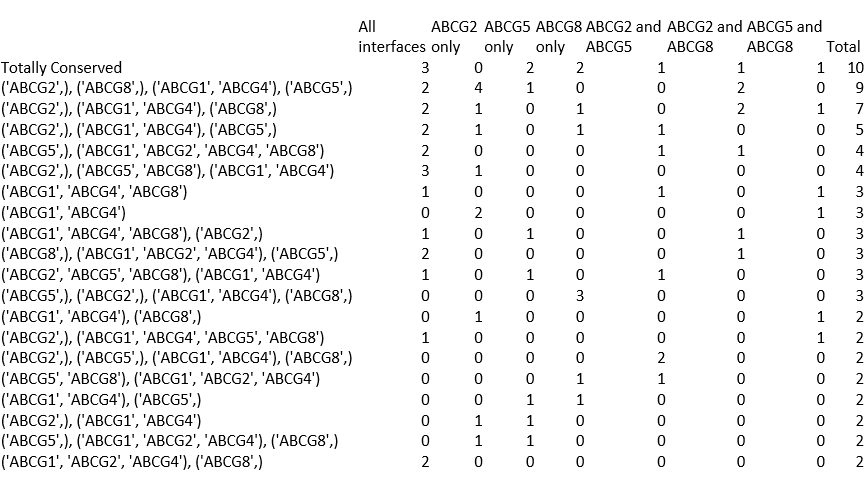


**Supplementary table 5: Frequency of conservation patterns at dimer interfaces.** 26 patterns with a single representative have been omitted for clarity


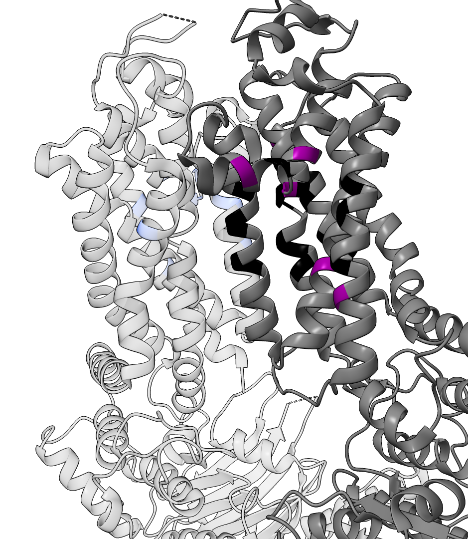

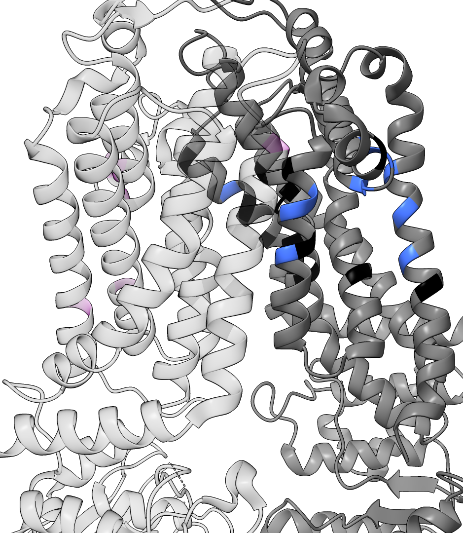

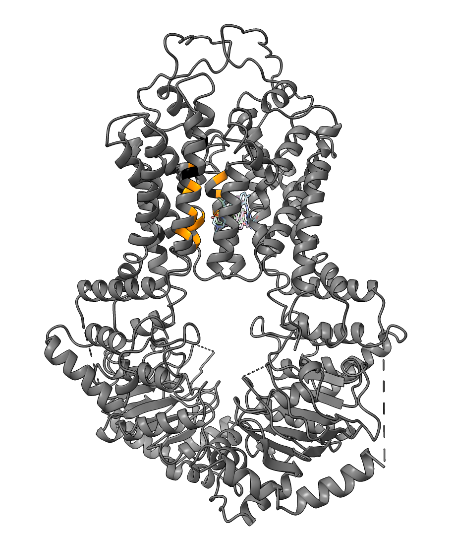

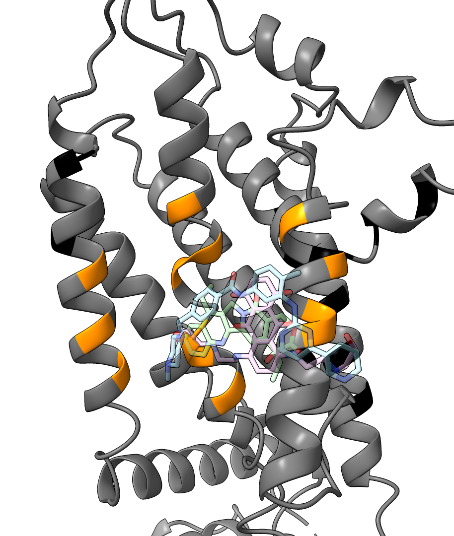


**T542**

**V546**


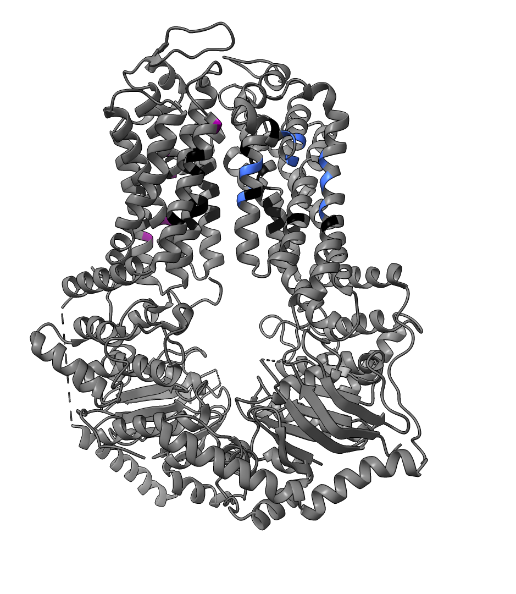


**L536**

**A540**

**Y570**

**T566**

**S594**

**W598**

**a)**

**i)**

**ii)**

**b)**

**i)**

**ii)**

**iii)**

**Supplementary Figure 4: Binding pockets of ABCG2, ABCG5 and ABCG8. a) ABCG2 (6VXF) from the side (i) and from the TMD-TMD interface (ii).** Binding pocket residues shown in orange, with the binding pockets of ABCG5 and ABCG8 in black. In **ii)**, one monomer has been removed for clarity. V546 and T542 align with the residues A540 and L536 in the binding pocket of ABCG5. Imatinib, Mitoxantrone and SN38 (from 6VXH, 6VXI, and 6VXJ, respectively) are shown as translucent.

**b) ABCG5/G8 from the side (i) and showing the binding pockets of ABCG5 (ii) and ABCG8 (iii).** ABCG5 binding pocket residues shown in blue, ABCG8 binding pocket residues shown in purple. Residues aligning to the binding pockets of the other proteins are shown in black. Residues Y570 and T566 in ABCG5 align to residues W598 and S594 in ABCG8. In **ii)** and **iii)** ABCG8 and ABCG5 are shown as translucent. These views have been rotated relative to **a) ii)** to show the relevant binding pockets more fully.


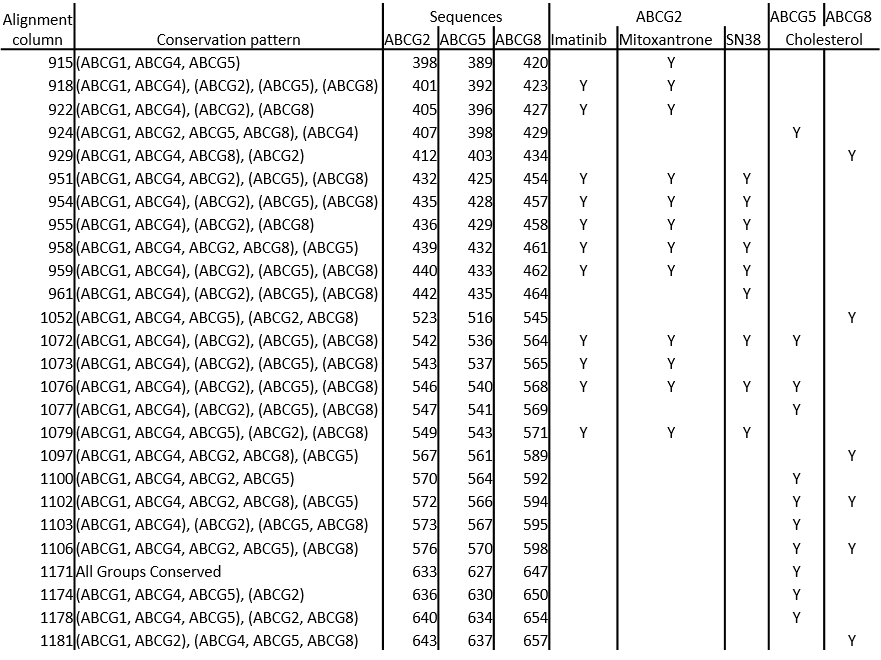


**Supplementary Table 6: Residues contributing to the binding pockets of ABCG2 and ABCG5/G8.** A column’s contribution to the binding pocket is indicated for ABCG2 (with Imatinib, Mitoxantrone, or SN38) or ABCG5/G8 as positive (Y) or negative (left blank).

**Supplementary Figure 5: Well populated Type II divergence patterns** shown as spheres. **a) Totally conserved b)** **(ABCG1, ABCG4), (ABCG2, ABCG5, ABCG8). c) (ABCG1, ABCG4, ABCG2), (ABCG5), (ABCG8)** One left out conservation: **d) (ABCG1, ABCG4, ABCG5, ABCG8), (ABCG2). e) (ABCG1, ABCG4, ABCG2, ABCG8), (ABCG5). f) (ABCG1, ABCG4, ABCG2, ABCG5), (ABCG8).** Analogous patterns for ABCG4 and ABCG1 have few members (5 and 0 respectively).


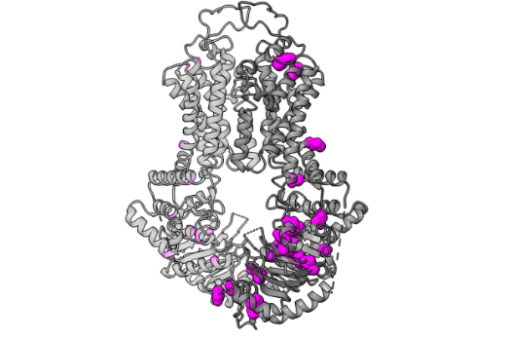

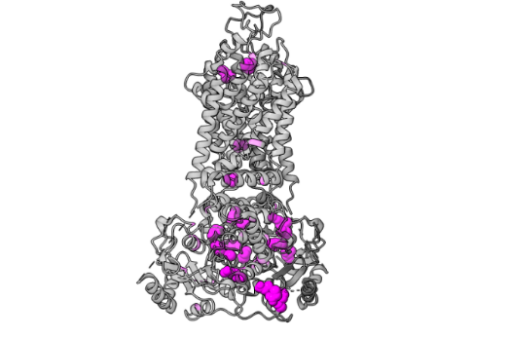

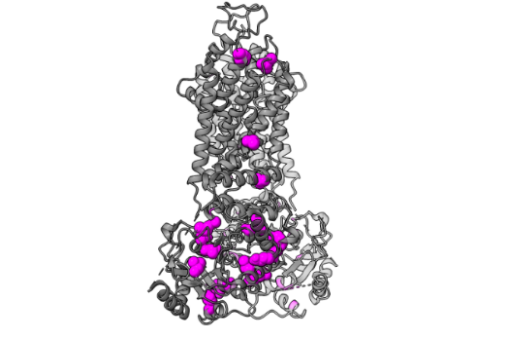

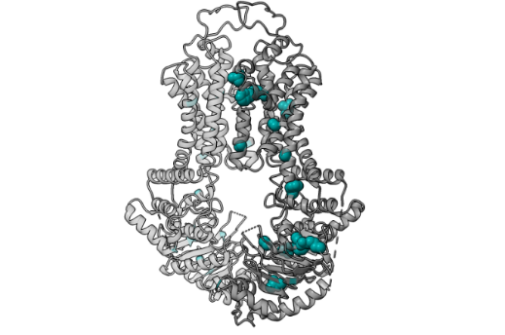

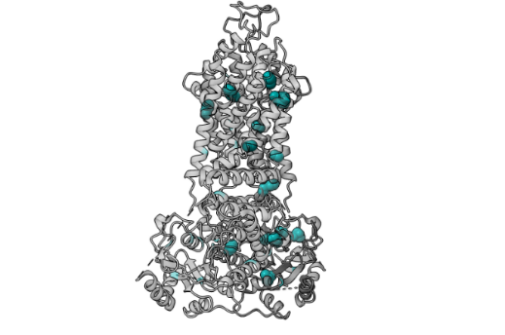

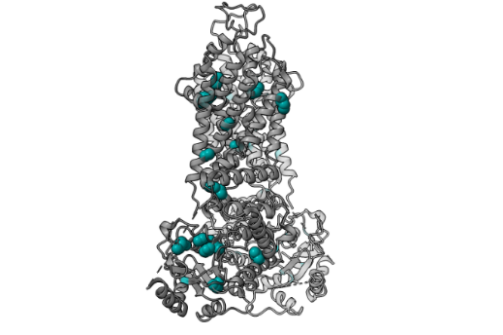

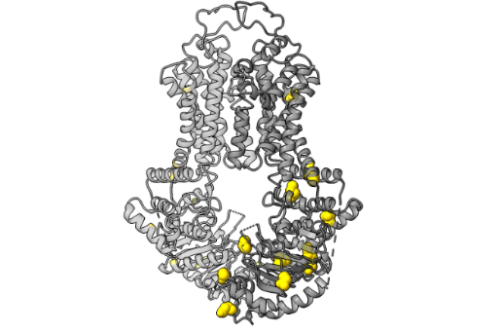

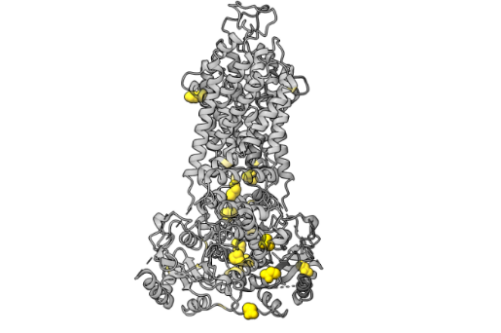

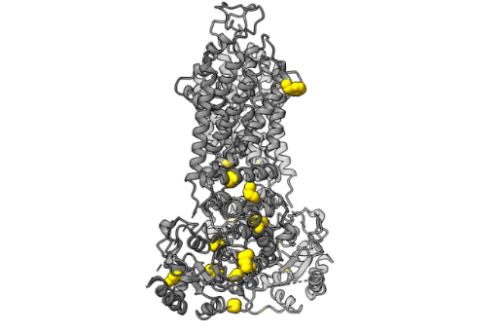

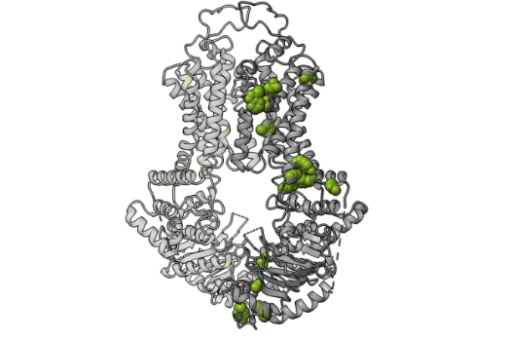

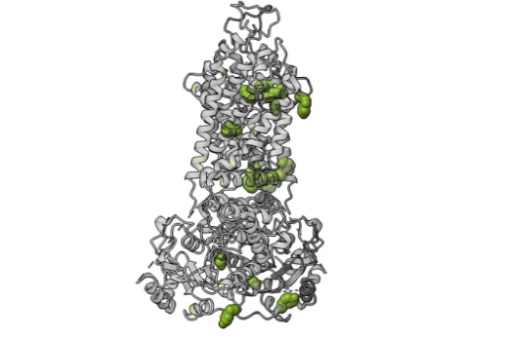

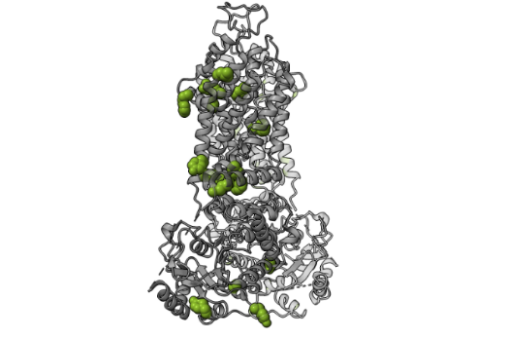

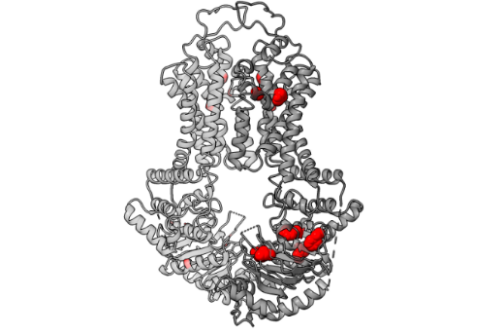

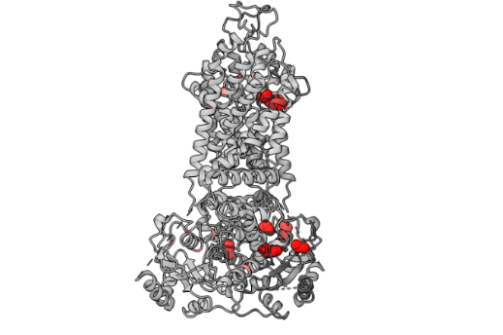

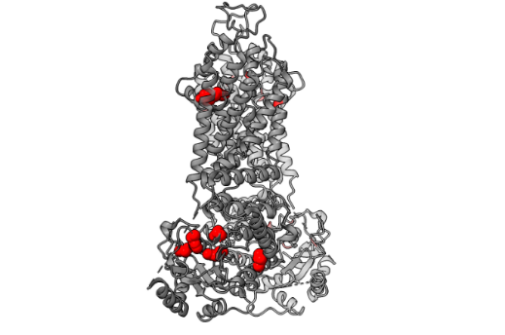


**a)**

**b)**

**c)**

**d)**

**e)**


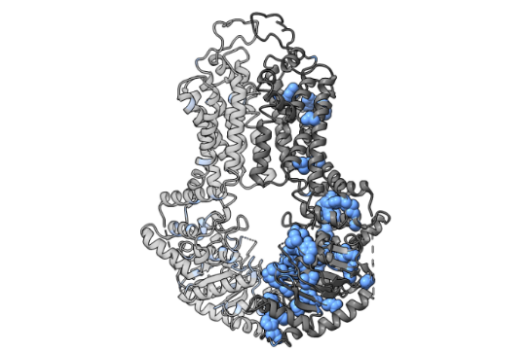

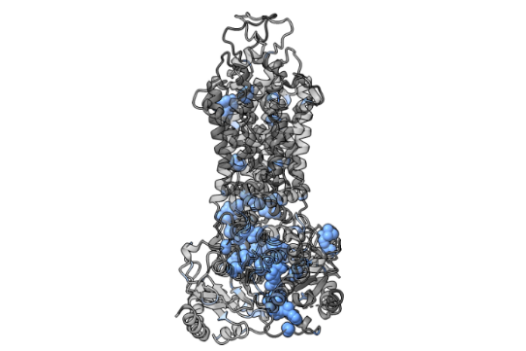

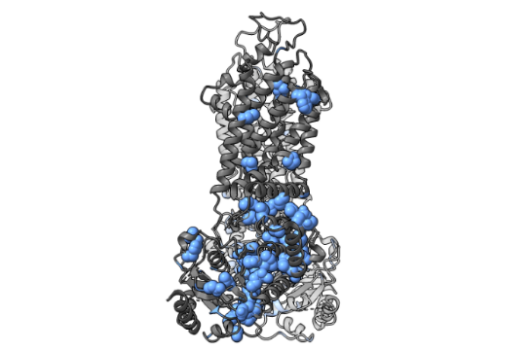


**f)**
